# Sleep duration of underserved minority children in a cross-sectional study

**DOI:** 10.1186/1471-2458-13-648

**Published:** 2013-07-12

**Authors:** William W Wong, Christina L Ortiz, Debra Lathan, Louis A Moore, Karen L Konzelmann, Anne L Adolph, E O’Brian Smith, Nancy F Butte

**Affiliations:** 1 USDA/ARS Children’s Nutrition Research Center, Department of Pediatrics, Baylor College of Medicine, Houston, Texas, USA; 2 Houston Parks and Recreation Department, Houston, Texas, USA

**Keywords:** Sleep, Minority children, Obesity

## Abstract

**Background:**

Short sleep duration has been shown to associate with increased risk of obesity. Childhood obesity is more prevalent among underserved minority children. The study measured the sleep duration of underserved minority children living in a large US urban environment using accelerometry and its relationship with BMI, socioeconomic status (SES), gender, ethnicity and physical activity.

**Methods:**

Time spent on sleep and physical activity among 333 Hispanic and 150 black children (9–12 y) was measured objectively by accelerometry over 5–7 consecutive days. The children were recruited at 14 underserved community centers in Houston, Texas, between January 2009 and February 2011. Body weight and height were measured in duplicate.

**Results:**

The majority of children (88.8%) wore the monitor for 6 consecutive days. The children slept 8.8 ± 0.6 (mean ± SD) h/d and spent 45 ± 24 min/d on moderate-vigorous physical activity (MVPA). Hispanic children slept 0.2 h/d longer (P < 0.001) than black children. Obese children slept 0.2 h/d less (P < 0.02) than normal-weight children. SES had no effect on sleep duration. There was a significant interaction between gender and age (P < 0.03); girls aged 11–12 y slept 0.3 h/d less than boys and the younger girls. Children slept 0.6 h/d longer (P < 0.001) during the weekend than weekdays. No relation was detected between sleep duration and MVPA time.

**Conclusions:**

Minority children living in a large metropolitan area in the US are not meeting the National Sleep Foundation recommendation for sleep duration of 10–11 h/d. Longitudinal studies based on objective measures are needed to establish causality between sleep duration and obesity risk among minority children.

## Background

The prevalence of sleep disturbances has increased dramatically, concurrent with the obesity epidemic in the US [1]. The National Sleep Foundation recommends that school-age children should sleep 10–11 h/d [2]. The recommendation was supported by observational data from two large cross-sectional studies involving thousands of children and a meta-analysis of sleep data based on 12 studies with 30,002 children aged 3–18 y from France, Tunisia, Japan, Germany, USA, Brazil, Portugal, United Kingdom, Canada, Taiwan and China [3-5]. Numerous studies also have documented that short sleep duration is associated with increased risk of childhood and adult obesity [3,4,6-10]. In an adult sleep debt study, short sleep duration led to a decrease in serum leptin and an increase in ghrelin suggesting that short sleep might stimulate appetite and increase food intake [11,12]. A recent study on 30 healthy adults also showed that sleep restriction led to increased activation of brain regions that are sensitive to food stimuli [13].

The latest National Health and Nutrition Examination Survey 2009-2010 showed that the prevalence of overweight and obesity was highest among Hispanic boys (39.6% overweight, 23.4% obese) and black girls (41.3% overweight, 24.3% obese), ages 2–19 y [14]. The sleep duration among underserved minority children is not well documented.

This study describes sleep duration and its association with BMI, socioeconomic status (SES), gender, ethnicity and physical activity in a large sample of underserved minority children living in an urban environment using an objective instrument, the Actical accelerometer.

## Methods

### Study population

A total of 483 minority children aged 9–12 y, regardless of body weight, with no physical or medical limitations to free play, and living in the Greater Houston Metropolitan area were enrolled in the Healthy Kids-Houston Study between January 2009 and February 2011. The Healthy Kids-Houston is a community-based after-school program to promote healthy lifestyle among minority children. Each program consists of three 6-week sessions, once in the fall, once in the spring and once at the end of the school year. The children were recruited from economically distressed neighborhoods surrounding 14 community centers managed by the City of Houston Parks and Recreation Department (HPARD) and are representative of minority children living in the Greater Houston Metropolitan Area. The program was promoted through newsletters of the community centers and at nearby public schools. These schools were populated primarily by minority children with the majority of them qualified for free or reduced cost school meal programs. The data collection was done over two years due to limited physical capacity of the community centers and to insure sufficient staff for program implementation and child safety. The study protocol was approved by the Institutional Review Board for Human Subject Research for Baylor College of Medicine (BCM) and Affiliated Hospitals. To enroll in the study, the parents completed the HPARD enrollment form and the BCM consent form. The program brochures, enrollment forms, and consent forms were available in both English and Spanish.

### Weight and height

Body weight and height were measured in duplicate with an electronic digital scale and with a digital stadiometer to the nearest 0.1 kg and 0.1 cm, respectively. The electronic scale and stadiometer were calibrated with a reference weight and rod, respectively, prior to the measurements. The measurements were made by project staff who had received training on the proper measurement procedures and the proper use of the equipment. Children were considered obese if their BMI values were ≥ 95^th^ percentile, overweight if their BMI values were ≥ 85^th^percentile but < 95^th^ percentile, and normal-weight if their BMI values were ≥ 5^th^ percentile but < 85^th^ percentile.

### Sleep duration

Actical (Philips Respironics, Bend, OR) accelerometer-based monitors were used to objectively measure sleep duration [15]. The monitors were affixed above the iliac crest of the right hip with an elastic belt and adjustable buckle. Children were instructed to wear the activity monitor for seven consecutive days, and to remove the monitor only for bathing or swimming. A log recording the times and reasons for monitor removal, nap and night sleep times was kept by the children and parents. Night sleep duration was determined from the 24-h accelerometer measurements by visual inspection by a single qualified technician in order to minimize inter-interpreter variation. A plot of activity counts per minute (cpm) for each 24-h period was used to identify the time of sleep onset and termination. The onset of night sleep was identified by a run-in period of consecutive zeros, followed by a longer period, usually lasting several hours, of consecutive zeros with occasional minute values with counts <200 cpm, indicative of movement during sleep. The termination of night sleep was identified by an abrupt increase in activity counts >200 cpm that lasted several minutes, indicative of awakening and getting out of bed. Any minutes scored >200 cpm during the night were considered awake and were removed from the sleep duration. The activity logs were used to confirm night sleep and to identify naps, which were uncommon in this group of children.

### Physical activities

The amount of time the children spent on sedentary, light and moderate-vigorous physical activities was also extracted from the Actical data [15]. Sedentary level was defined as activity energy expenditure (AEE) <0.01 kcal^.^kg^-1.^min^-1^ or physical activity ratio (PAR) < 1.5, encompassing physical activities of minimal body movements in the sitting or reclined position. Light level was set at 0.01 < AEE < 0.04 kcal^.^kg^-1.^min^-1^ or 1.5 < PAR < 3.0, reflective of a low level of exertion in the standing position. Moderate level was set at 0.04 < AEE <0.10 kcal^.^kg^-1.^min^-1^ or 3.0 < PAR < 6.0, and involved medium exertion in the standing position. Vigorous level was set at AEE > 0.10 kcal^.^kg^-1.^min^-1^ or PAR > 6.0, reflective of activities at a high level of exertion in the standing position.

### Socio economic status (SES)

The SES of the children was based on information provided by the parents in the program enrollment form. Children were considered to be from low socioeconomic families when they qualified for free or reduced cost meal program at school and qualified for federal or state medical insurance programs for low-income families.

### Statistical procedures

Descriptive statistics were used to generate the means and standard deviations of all the outcome measures. Independent-samples t-test or Pearson Chi-Square test was used to evaluate potential differences between the race/ethnicity groups. Generalized linear models including all 2- and 3-way interactions were used to test the effects of gender, age (9–10 y *vs.* 11–12 y), race/ethnicity (black *vs*. Hispanic), obesity status (normal-weight, overweight, obese) and SES on sleep duration with and without community centers in the model. Non-significant 2- and 3-way interactions, starting with the most non-significant 3-way interaction, were removed from the model one at a time using a backward stepwise elimination procedure. The same statistical procedure was used to examine the relationship between sleep duration and physical activity. Generalized estimating equations for repeated measures was used to test the potential differences in sleep duration between weekdays and weekend days with adjustment for age, gender, race/ethnicity, obesity status and community center. Statistical analyses were performed with SPSS software (version 20, SPSS Inc, Chicago, IL). An alpha value of 0.05 was used in all statistical analyses.

## Results

Table 1 describes the demographic and physical characteristics of the 483 children. Age, gender distribution, and body weight did not differ by race/ethnicity (P = 0.35). The black children were taller (P = 0.001) and the Hispanic children were heavier by body mass index (BMI, P < 0.001) and BMI z-score (P < 0.001). The majority of children (66.1%) were overweight or obese with more Hispanic children falling into these categories (76.9% vs. 42.0%, P < 0.001) than black. Approximately 93% of the children were from low-income families with more Hispanic children falling into the low SES category (P < 0.006) than black.

**Table 1:** **Demographic and physical characteristics of study subjects**^**a**^

**Variables**	**All children**	**Hispanics**	**Black**
N	483	333	150
Age, y	10.3 ± 0.99^b^	10.3 ± 0.99	10.4 ± 0.99
Gender (M/F)	247/236	175/158	72/78
Weight, kg	47.6 ± 16.5	48.5 ± 15.2	45.7 ± 19.0
Height, cm	141.9 ± 9.2	141.0 ± 9.1	144.0 ± 9.2*
BMI, kg/m^2^	23.3 ± 6.1	24.0 ± 5.4	21.6 ± 7.1**
BMI z-score	1.32 ± 1.03	1.54 ± 0.87	0.82 ± 1.18**
Weight status, N (%)			
Normal	164 (34.0)	77 (23.1)	87 (58)**
Overweight	84 (17.4)	66 (19.8)	18 (12.0)**
Obese	235 (48.7)	190 (57.1)	45 (30.0)**
Low SES (%)^c^	93.2	95.5	88.4*

^a^ Hispanic and black groups were compared using independent-samples t-test for continuous variables and Pearson Chi-Square for categorical variables. Significant differences between Hispanic and black are indicated as follows: * for P < 0.01 and ** for P < 0.001.

^b^ Mean ± SD.

^c^ SES = socio economic status. Based on qualification for free/reduced cost meals at school and Medicaid.

**Figure 1:**
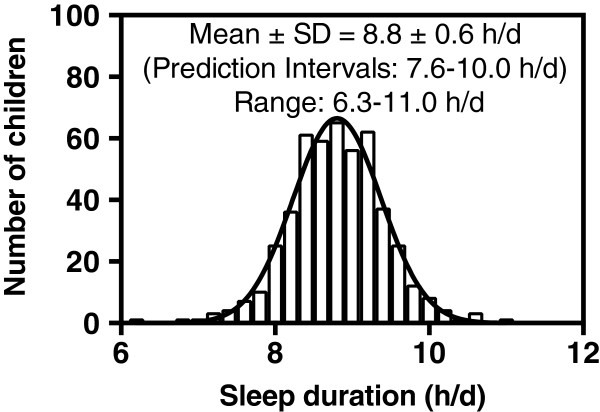
**Frequency distribution of sleep duration in 483 underserved minority children.** The histogram represents the sleep duration per day for the children in a given interval.

The majority of children (88.8%) wore the Actical for at least 6 consecutive days while 72.9% wore the Actical for 7 consecutive days. Figure 1 shows the distribution of sleep duration recorded for all the children. The children slept an average of 8.8 ± 0.6 h/d (Prediction Intervals: 7.6-10.0 h/d). Among the 483 children, only 12 children slept 10 hours or longer.

**Figure 2:**
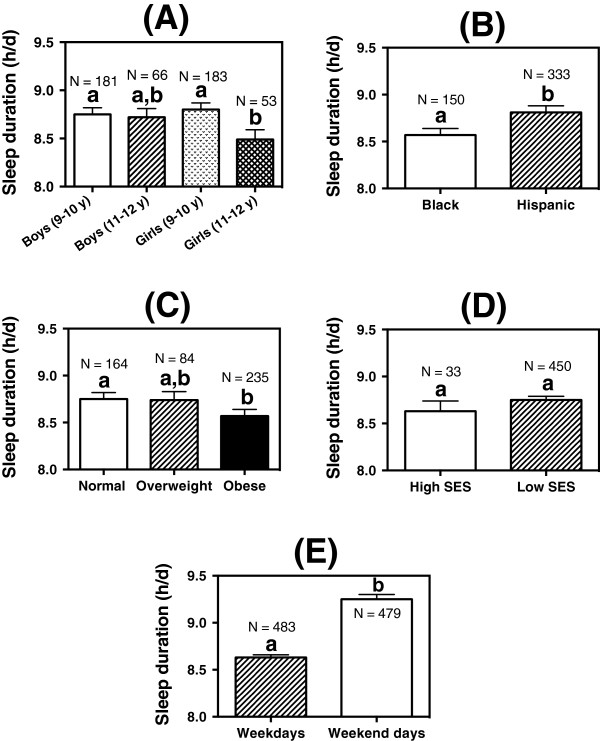
**Estimated sleep duration (h/d). (A)** gender and age: Boys 9–10 y, n = 181; Boys 11–12 y, n = 66; Girls 9–10 y, n = 183; Girls 11–12 y, n = 53. **(B)** race/ethnicity: Black, n = 150; Hispanic, n = 333. **(C)** obesity status: Normal, n = 164; Overweight, n = 84; Obese, n = 235. **(D)** socioeconomic status (SES): High SES, n = 33; Low SES, n = 450. **(E)** weekend/weekday: Weekdays, n = 483; Weekend days, n = 479. The whisker represents the standard error of the mean. Different letters (a, b) above the columns in each figure indicate a significant difference.

Figure 2 shows the effects of gender, age, race/ethnicity, obesity status, SES, and weekday/weekend on sleep duration based on a generalized linear model. A significant interaction was detected between gender and age (P = 0.03). As shown in Figure 2A, the older girls (11–12 y) slept less (−0.2 to −0.3 h/d, P < 0.03) than the younger children (9–10 y). The Hispanic children slept more (+0.2 h/d, P <0.001) than the black children (Figure 2B). Obese children slept less (−0.2 h/d, P = 0.02) than the normal-weight children (Figure 2C). SES had no effect on sleep duration (Figure 2D, P = 0.26). Inclusion of community center in the analysis did not change the outcomes as reported in Figures 2A, B, C and D. Figure 2E shows that children slept more (+0.6 h/d, P < 0.001) during weekend than weekdays. The results remained unchanged with the inclusion of age, gender, race/ethnicity, obesity status and community center in the model. When obesity status was replaced by BMI z-scores in the generalized linear model, sleep duration was reduced by 0.1 h/d (P = 0.001) with each unit increase in BMI z-score. Since the majority of the children (93%) were from low-income families, the generalized linear model analysis was repeated without adjusting for SES. Similar results were obtained for race/ethnicity (Hispanic children slept 0.3 h/d longer than black children, P < 0.001), obesity status (obese children slept 0.2 h/d less than normal-weight children, P < 0.01), and gender/age (older girls slept 0.3 h/d less than the younger girls and boys, P < 0.02). Similar results also were obtained when we limited the statistical analysis on the children who wore the Actical for 7 consecutive days.

Overall, the children spent 528 ± 63 min/d on sedentary physical activity, 281 ± 57 min/d on light physical activity but only 45 ± 24 min/d on moderate-vigorous physical activity (MVPA). The amount of time the children spent on MVPA was less than the US public health recommended level of 60 min/d [16]. Generalized linear models analysis with adjustment for age, gender, race/ethnicity, obesity status and center showed no relationship between MVPA and sleep duration (P = 0.27). The same analysis, however, showed a significant negative relationship between sedentary/light physical activities and sleep duration (P < 0.01)

## Discussion

The majority of the sleep studies in children are based on subjective reports from parents. Only four studies reported the use of accelerometry to measure sleep duration in children [17-20]. The study reported by Gupta was done on adolescent girls aged 11–16 y based on 24-h of wrist actigraphy [19]. Benefice reported the sleep duration on Senegalese malnourished adolescent girls aged 13.3 y based on 3–4 d of accelerometry measurements [20]. Nixon measured the sleep duration on 7-y-old children by wrist actigraphy [18]. The most recent study measured the sleep duration of 308 children aged 4–10 y using wrist actigraphy over one week with the majority (71.4%) of the children being white [17]. By convention, wrist actigraphy has been used in sleep studies. However, as demonstrated in a recent publication comparing sleep duration measured by wrist- or hip-accelerometers in 10–11 y old children, both accelerometers yielded identical results and the difference of 6.8 minutes was considered to be clinically non-significant [21]. The accuracy of sleep duration measured by Actical accelerometers is further supported in a separate study on 30 adolescents who wore three actigraphs (Actical, Sleepwatch and Actiwatch) concurrently while their sleep duration was assessed by the reference method, polysomnography [22]. The study showed that Actical yielded similar sleep duration when compared to Sleepwatch and Actiwatch. Interestingly, all three actigraphs overestimated sleep duration by 25–31 min/d when compared to polysomnography during a single night measurement. The authors indicated that the difference might be due to the wake-time sensitivity between actigraph and polysomnography. However, if the overestimation of sleep duration is correct, we would have overestimated the sleep duration of the underserved minority children and therefore they would be in further sleep deficit. The agreement in sleep duration measurements between Actical and Actiwatch has also been demonstrated in 20 young adults who wore both monitors for 5–9 consecutive days [23]. Polysomnography certainly is the reference method to measure sleep duration. However, the procedure is expensive and invasive because it requires the participants to sleep in a sleep laboratory and therefore removing them from their natural sleeping settings. Furthermore, polysomnography will most likely limited the measurement to a single night whereas accelerometers such as Actical will allow measurement of sleep duration over a period of several days and therefore provide a more representative measurement of sleep duration. It also will be difficult to implement the polysomnography procedure in a study involving large samples of children. Therefore this study represents the first objective measurement of sleep duration over a period of 5–7 d of a large sample of underserved minority children aged 9–12 y living in a large US urban and economically distressed environment.

Our results showed that these underserved minority children were not meeting the National Sleep Foundation recommended sleep duration of 10–11 h/d for school-aged children [2]. Our results (Figure 2A) showed that older girls slept less than the younger children and that black children also slept less than Hispanic children (Figure 2B). If short sleep duration is a risk factor for childhood obesity, older black girls should be encouraged to sleep longer because they have been documented to have higher prevalence of overweight and obesity than black boys and Hispanic girls [14]. Since obese children slept less than normal-weight children (Figure 2C), parents should encourage their children to go to sleep earlier or to provide a home environment to encourage uninterrupted sleep. We found no association between SES and sleep duration (Figure 2D). Since the project targeted the underserved communities, the lack of association between SES and sleep duration is probably attributable to the small number of children (n = 33) in the high SES category. As shown earlier, excluding SES in the analysis did not affect the effects of BMI, race/ethnicity and age/gender on sleep duration.

In Houston, Hispanics and blacks account for ~62% of the population. More importantly, Hispanics and blacks constitute at least 30% of the population in the United States’ most populous states such as California, Texas, New York, Florida, Illinois, Arizona, New Mexico, New Jersey, Nevada and North Carolina. Since these states accounted for almost 50% of the total U.S. population, obesity-related diseases, particularly among the minority populations, will have a major negative impact on the local, state, and federal healthcare system. Our sleep data showed that minority children, including the normal-weight children, were not meeting the recommended sleep duration of 10–11 h/d. Our results also showed that both BMI z-scores and obesity status of the minority children were negatively associated (P < 0.01) with sleep duration suggesting that longer sleep duration might lead to an improvement in BMI z-scores and obesity status. Our cross-sectional data cannot substantiate the causal relationship which would require longitudinal observations. Our results also showed that only 12 of the 483 minority children were sleeping 10 h/d or longer. With most minority populations living in the major U.S. cities, it is likely that the demographic profile of the minority population living in Houston, Texas might be similar to those living in the other major metropolitan cities. If lack of sleep is a risk factor for childhood obesity, families should encourage their children to go to bed earlier in order to meet the sleep recommendation.

In the most recent national survey in the United States [14], the prevalence of overweight and obesity remained significantly elevated among minority children when compared to white children. The survey showed that approximately 25% of white children were overweight and between 6% and 15% were obese depending on their ages. A recent study measured the sleep duration on 308 children (7.2 ± 1.3 y) over a 1-wk period using wrist accelerometry and found that these children slept an average of 8 h/d [17]. Since the majority of the children (71.4%) in the study was white, together with our data on minority children, one can speculate that children living in the United States, regardless of race/ethnicity, might not be meeting the National Sleep Foundation recommendation of 10–11 h/d of sleep.

There is a possibility that children might take daytime naps and compensate for the short sleep duration, particularly among the Hispanic children. However, children between 9 and 12 years of age seldom take daytime naps, particularly during school days. In an unrelated study of 4,470 Chinese children living in Hong Kong with an average age of 9.2 ± 1.8 y [24], only approximately 6% of the children took naps. Therefore, it is not likely that daytime naps among our underserved minority children will compensate for their short sleep duration.

In a cross-sectional study of 5,159 Chinese children with an average age of 9.3 ± 1.8 y, children who slept more during the weekend had lower risk of overweight and obesity [25]. The sleep data were collected using a 54-item sleep questionnaire and the anthropometric data were based on parent reports. When weekend sleep compensation was included in the generalized linear models, we found no compensation effect on BMI z-scores or obesity status (P ≥ 0.18). The much larger sample size in the study of Chinese children might increase the power to detect the effect of sleep compensation on obesity risk. It is also possible that there is a significant race/ethnicity difference in sleep compensation between Chinese children and Hispanic and black children. To evaluate the potential compensation effect, a much larger sleep study among underserved minority children is needed. However, if the sleep compensation result reported among the Chinese children is true and applicable to other race/ethnic groups, the result indirectly suggested that the 10–11 h/d sleep duration recommended by the National Sleep Foundation might be appropriate*.*

In a study carried out in Hong Kong, leisure extracurricular activities and later school start time were found to lengthen sleep time among school-aged children [24]. However, high socioeconomic status, media use and homework were found to shorten sleep time. In a separate study among adolescents [26], a delay of school start time by 30 minutes led to an increase in sleep time by 45 minutes; more satisfaction with sleep; improved motivation; reduced sleepiness, fatigue and depressed mood; and improved class attendance. Both studies again suggested that longer sleep duration might have many beneficial effects including reducing the risk of obesity. The beneficial effects by delaying school start time are very interesting and need to be confirmed, particularly among underserved minority children. With schools pushing for better academic outcomes, children are often given more homework. If the negative effect of homework on sleep time is real, it will be a challenge for the schools to balance between school hours and homework load. With the ready accessibility of television, computer, and other digital devices in most families, programs to promote a healthy lifestyle by reducing screen and video game time are needed because media use was found to shorten sleep time*.*

In a cross-sectional study of 4,511 Portugese children aged 7–9 y based on questionnaires filled out by parents, children who engaged in physical activity slept more than sedentary children [4]. Therefore, physical activity might have a positive effect on sleep duration. However, based on accelerometry, we found a negative association between sedentary/light physical activities and sleep duration (P < 0.01). The negative association remained significant when times spent on sedentary and light physical activities were analyzed separately while controlling for age, gender, race/ethnicity, obesity status and center. No association (P = 0.27) was detected between sleep duration and time spent on MVPA among our minority children. The lack of association might be because of the low MVPA among these minority children [16]. To combat childhood obesity, children are encouraged to engage in 60 minutes or more per day of MVPA [27-29]. Therefore, the relationship between MVPA and sleep duration deserves more attention.

As indicated in the method section, if accelerometer counts are equal to zero, it is not possible to distinguish quiet time (e.g. lying motionless down quietly to listen to music) from actual sleep time. Therefore, inclusion of a quiet period prior to night sleep would artificially inflate the sleep time suggesting that the true sleep time might actually be even shorter than what we have reported.

Several meta-analyses documented that short sleep duration is associated with childhood and adult obesity [6-9]. A longitudinal study on children also showed that shorter sleep duration at 9 y of age was associated with an increased risk of obesity at 12 y of age [10].

There are limitations to our findings. First of all, this is a cross-sectional study without a control group. Therefore the results cannot be used to examine a causal effect of short sleep duration on obesity risk or to determine whether the underserved minority children were more vulnerable to sleep deprivation. However, accelerometry-determined sleep data on white children, presumably from higher income families, also showed that these children were not meeting the sleep recommendation suggesting that sleep deprivation might be a universal risk factor among all contemporary US children. Secondly, the sleep data were collected from children living in a southern US metropolitan city and therefore might not be applicable to underserved minority children living in other large metropolitan cities or suburban and rural environments. More studies using objective measures of sleep duration are needed to confirm and expand our findings in other regions in the US among all children.

## Conclusions

This study presents an objective assessment of sleep duration in a large sample of underserved minority children living in a large US metropolitan area. In agreement with the findings from the National Institute of Child Health and Human Development Study of Early Child Care and Youth Development on predominantly white children and a survey by the National Sleep Foundation [5,30], underserved black and Hispanic children are sleeping less than 9 h/d on average. Short sleep duration has been associated with increased risk of obesity [5,31,32] and adverse metabolic outcomes [17,33-35]. Large-scale longitudinal studies using objective measures are needed in order to establish causality between sleep duration and obesity risk and obesity-related co-morbidities among children.

## Abbreviations

AEE: Activity energy expenditure; BCM: Baylor College of Medicine; CPM: Counts per minute; HPARD: City of Houston Parks and Recreation Department; MVPA: Moderate-vigorous physical activity; PAR: Physical activity ratio; SES: Socioeconomic status.

## Competing interests

The authors declared that they have no competing interests. The contents of this publication do not necessarily reflect the views or policies of the U.S. Department of Agriculture or mention of trade names, commercial products, or organizations imply endorsement.

## Author information

WWW is the Project Director of the Healthy Kids-Houston project and a Professor of Pediatrics at BCM. CLO is the Principal Investigator of the Healthy Kids-Houston project and an Administrator Manager at HPARD. DL is the Assistant Director at HPARD. LAM is a Co-Investigator of the Healthy Kids-Houston project and a Senior Superintendent at HPARD. KLK is a Project Consultant of the Healthy Kids-Houston project. ALA is an Electronic Engineer at the USDA/ARS Children’s Nutrition Research Center. EOS is the Biostatistician of the Healthy Kids-Houston project and a Professor of Pediatrics at BCM. NFB is a Professor of Pediatrics at BCM and the Director of the Energy Metabolism Laboratory at the USDA/ARS Children’s Nutrition Research Center.

## Authors’ contributions

WWW was responsible for the implementation of the Healthy Kids-Houston that generated the sleep data for the manuscript. CLO, DL and LAM identified the community centers to support the project. They also provided the instructors to help collect the data. KLK assisted in subject recruitment. ALA and NFB assisted in the placement of the Acticals on the children and on converting the Actical data into activity times and sleep duration. EOS assisted in the study design and the statistical analysis of the sleep data. All the authors were involved in the original study design, assisted in the implementation of the project, and read and approved the final manuscript.

## Pre-publication history

The pre-publication history for this paper can be accessed here:

http://www.biomedcentral.com/1471-2458/13/648/prepub
